# Supplementary material for: Prediction of In-Hospital Falls Using NRS, PACD Score and FallRS: A Retrospective Cohort Study
Source: Geriatrics (Basel). 2023 Jun 1;8(3):60. doi: 10.3390/geriatrics8030060 (PMC10298410; doi:10.3390/geriatrics8030060)
Supplement: Supplementary file 1 [file geriatrics-08-00060-s001.zip › geriatrics-2343948-supplementary.pdf]

# Prediction of In-Hospital Falls Using NRS, PACD Score and FallRS: A Retrospective Cohort Study

**Jennifer Siegwart [1,*,†], Umberto Spennato [1,†], Nathalie Lerjen [1], Beat Mueller [1,2], Philipp Schuetz [1,2], Daniel Koch [1,‡] and Tristan Struja [1,‡]**

1 Medical University Clinic, Kantonsspital Aarau, 5001 Aarau, Switzerland; schuetzph@gmail.com (P.S.)
2 Medical Faculty, University of Basel, 4001 Basel, Switzerland
* Correspondence: jennifer.siegwart@gmail.com
† These authors contributed equally to this work.
‡ These authors contributed equally to this work.

VERSION: May8th 2023

SUPPLEMENTARY MATERIAL: 11 tables, 2 figures

**Corresponding author and person to whom reprint requests should be addressed:**

Jennifer Siegwart
Medical University Clinic
Kantonsspital Aarau
Tellstrasse 25
CH-5001 Aarau, Switzerland
jennifer.siegwart@gmail.com

**Table S1 Composition of the NRS as presented in the original study (Kondrup 2003)**

| **Nutritional status** | | Score |
|---|---|---|
| Normal | - | 0 |
| Mild | weight loss > 5% in 3 months<br>OR<br>food intake below 50-75% of requirement in preceding week | 1 |
| Moderate | weight loss > 5% in 2 months<br>OR<br>food intake below 25-50% of requirement in preceding week<br>OR<br>BMI 18.5-20.5 | 2 |
| Severe | weight loss > 5% in 1 month<br>OR<br>food intake below 0-25% of requirement in preceding week<br>OR<br>BMI < 18.5 | 3 |
| **Severity of disease** | | |
| Normal | - | 0 |
| Mild | e.g. hip fracture, diabetes | 1 |
| Moderate | e.g. pneumonia | 2 |
| Severe | e.g. head injury, intensive care patients | 3 |
| **Age** | >70 years | 1 |

Abbr: NRS (Nutritional Risk Score), BMI (Body Mass Index)
Legend: Add the Scroe points for a maximum score of 7

**Table S2 Composition of PACD as presented in the original study (Conca, Gabele et al. 2018)**

| | |
|---|---|
| Number of active medical problems on admission | Infinite |
| Person in the same household who can provide help<br>Yes = 0 points, No = 4 points | Max. 4 points |
| Number of limitations in ADL (1 point per limitation)<br>Needed help for:<br>- Personal hygiene<br>- Dressing/undressing<br>- Toileting<br>- Bathing/showering<br>- Eating/drinking<br>- Moving<br>- Transfer (bed/chair)<br>- Car or public transportation<br>- Shopping<br>- Cooking<br>- Homework<br>- Medication management | Max. 12 points |
| Age (years)<br><60 = 0 points<br>≥60 = 1 point<br>≥70 = 2 points<br>≥80 = 3 points<br>≥90 = 4 points<br>≥100 = 5 points | Max. 5 points |

Abbr: PACD (Post Acute Care Discharge Score), ADL (Activities of Daily Living)
Legend: Add all points for the score of your patient. Because there is no maximum number of medical problems, the score potentially ranges to infinity.

**Table S3 Composition of DOS as presented in the original study (Marieke J Schuurmans 2003)**

| Observation | No (points) | Yes (points) |
|---|---|---|
| Falls asleep during conversation | 0 | 1 |
| Quickly distracted by environmental stimuli | 0 | 1 |
| Remains attentive to the conversation or the action | 1 | 0 |
| Does not finish questions or answers | 0 | 1 |
| gives inappropriate responses to questions | 0 | 1 |
| Reacts slowly to orders | 0 | 1 |
| Missing local orientation | 0 | 1 |
| Knows daytime | 1 | 0 |
| Remembers recent events | 1 | 0 |
| Restlessness | 0 | 1 |
| Pulls on installations | 0 | 1 |
| Reacts inappropriate emotional | 0 | 1 |
| Visual, auditory or olfactory hallucinations | 0 | 1 |

Abbr: DOS (Delirium Observation Screening Scale)
Legend: Add the points for a maximum score of 13.

**Table S4 Predictive ability for in-hospital fall of DOS. Derivation years 2016 – 2019 and validation years 2020 – 2022.**

| | **Derivation 2016-2019 AUC (95% CI)** | **Validation 2020-2022 AUC (95% CI)** | **P-value** |
|---|---|---|---|
| **DOS** | 0. 58 (0.48 – 0.67) | 0.63 (0.60 – 0.67) | 0.04 |

Abbr: AUC (Area under curve), CI (confidence interval), DOS (delirium observation screening scale)

**Table S5 Calibration for NRS (nutritional risk screening)**

| NRS | Probability (%) | Observed Falls | Expected Falls | Total Sample |
|---|---|---|---|---|
| 0 | 1.8 | 6 | 12.8 | 706 |
| 1 | 2.3 | 31 | 35.4 | 1,517 |
| 2 | 3.0 | 60 | 49.3 | 1,649 |
| 3 | 3.8 | 40 | 34.1 | 891 |
| 4 | 4.9 | 31 | 25.9 | 530 |
| 5 | 6.2 | 13 | 20.8 | 335 |
| 6 | 7.9 | 5 | 7.6 | 97 |
| 7 | 9.9 | 1 | 1.1 | 11 |

**Table S6 Calibration for DOS (delirium observation screening scale)**

| DOS | Probability (%) | Observed Falls | Expected Falls | Total Sample |
|---|---|---|---|---|
| 1 | 3.0 | 102 | 120.4 | 4,080 |
| 2 | 3.5 | 24 | 18.1 | 511 |
| 3 | 4.2 | 25 | 14.8 | 351 |
| 4 | 5.0 | 16 | 9.9 | 196 |
| 5 | 6.0 | 11 | 9.4 | 157 |
| 6 | 7.2 | 11 | 9.4 | 132 |
| 7 | 8.5 | 9 | 8.4 | 99 |
| 8 | 10.0 | 5 | 8.5 | 85 |
| 9 | 11.9 | 4 | 6.8 | 57 |
| 10 | 13.9 | 7 | 7.1 | 51 |
| 11 | 16.3 | 5 | 4.1 | 25 |
| 12 | 19.0 | 1 | 2.3 | 12 |
| 13 | 22.0 | 1 | 1.5 | 7 |
| 14 | 25.4 | 0 | 0.3 | 1 |

**Table S7 Calibration for FallRS (Fall Risk Score)**

| FallRS | Probability % | Observed Falls | Expected Falls | Total Sample |
|---|---|---|---|---|
| 0 | 1.3 | 0 | 0.0 | 2 |
| 1 | 1.5 | 0 | 0.9 | 64 |
| 2 | 1.6 | 0 | 2.6 | 159 |
| 3 | 1.8 | 3 | 2.5 | 139 |
| 4 | 2.0 | 1 | 2.8 | 139 |
| 5 | 2.2 | 5 | 3.9 | 176 |
| 6 | 2.4 | 4 | 4.8 | 199 |
| 7 | 2.7 | 5 | 4.5 | 166 |
| 8 | 3.0 | 5 | 5.1 | 170 |
| 9 | 3.3 | 6 | 4.2 | 127 |
| 10 | 3.7 | 4 | 4.4 | 121 |
| 11 | 4.0 | 7 | 4.0 | 98 |
| 12 | 4.5 | 5 | 4.2 | 95 |
| 13 | 4.9 | 5 | 3.7 | 74 |
| 14 | 5.5 | 3 | 2.8 | 52 |
| 15 | 6.0 | 5 | 4.3 | 71 |
| 16 | 6.6 | 4 | 3.7 | 55 |
| 17 | 7.3 | 2 | 3.7 | 50 |
| 18 | 8.1 | 6 | 3.0 | 37 |
| 19 | 8.9 | 2 | 2.1 | 24 |
| 20 | 9.8 | 2 | 4.0 | 41 |
| 21 | 10.7 | 1 | 3.0 | 28 |
| 22 | 11.8 | 2 | 2.0 | 17 |
| 23 | 12.9 | 2 | 1.9 | 15 |
| 24 | 14.1 | 2 | 1.7 | 12 |
| 25 | 15.5 | 1 | 1.2 | 8 |
| 26 | 16.9 | 0 | 1.7 | 10 |
| 27 | 18.4 | 1 | 0.6 | 3 |
| 28 | 20.0 | 0 | 0.2 | 1 |
| 29 | 21.7 | 1 | 0.7 | 3 |

**Table S8 Calibration for PACD (Post-Acute Care Discharge score)**

| PACD | Probability (%) | Observed Falls | Expected Falls | Total Sample |
|---|---|---|---|---|
| 0 | 1.6 | 0 | 0.0 | 3 |
| 1 | 1.7 | 2 | 4.8 | 281 |
| 2 | 1.9 | 4 | 5.5 | 290 |
| 3 | 2.1 | 2 | 6.6 | 314 |
| 4 | 2.3 | 10 | 6.5 | 283 |
| 5 | 2.6 | 5 | 6.3 | 246 |
| 6 | 2.8 | 7 | 5.1 | 182 |
| 7 | 3.1 | 5 | 4.9 | 156 |
| 8 | 3.4 | 9 | 5.1 | 150 |
| 9 | 3.8 | 7 | 4.3 | 115 |
| 10 | 4.2 | 6 | 5.2 | 124 |
| 11 | 4.6 | 5 | 3.6 | 79 |
| 12 | 5.1 | 5 | 4.9 | 96 |
| 13 | 5.6 | 4 | 4.1 | 73 |
| 14 | 6.1 | 5 | 3.9 | 64 |
| 15 | 6.7 | 3 | 4.3 | 64 |
| 16 | 7.4 | 6 | 4.1 | 56 |
| 17 | 8.1 | 4 | 4.8 | 59 |
| 18 | 8.9 | 2 | 3.1 | 35 |
| 19 | 9.8 | 1 | 3.1 | 32 |
| 20 | 10.7 | 1 | 2.6 | 24 |
| 21 | 11.7 | 1 | 2.0 | 17 |
| 22 | 12.8 | 2 | 0.9 | 7 |
| 23 | 14.0 | 0 | 0.7 | 5 |
| 24 | 15.3 | 0 | 0.5 | 3 |
| 25 | 16.6 | 2 | 1.0 | 6 |

**Table S9 Final multivariable model for NRS from validation data from 2018-2020**

Probability = $\frac{e^{(\beta 0+\ \beta_1 * var1+\beta_2 * var2+etc)}}{1+e^{(\beta 0+\ \beta x * var1+Bx2 * var2+etc)}}$

| Fall and NRS | Beta-Coefficient | OR | 95% Conf. Interval | |
|---|---|---|---|---|
| **NRS** | 0.09997 | 1.105137 | 0.9547286 | 1.27924 |
| **Age** | 0.01669 | 1.016835 | 0.9981085 | 1.035913 |
| **Elixhauser comorbidity index** | 0.16086 | 1.174522 | 1.027319 | 1.342818 |
| **Frailty score** | 0.11456 | 1.121378 | 1.061937 | 1.184146 |
| **Gender** | -0.69627 | 0.498439 | 0.3123563 | 0.7953785 |
| **Intercept** | -5.78050 | 0.0030872 | 0.0008177 | 0.0116554 |

**Table S10 Final multivariable model for PACD from validation data from 2018-2020**

| Fall and PACD | Beta-Coefficient | OR | 95% Conf. Interval | |
|---|---|---|---|---|
| **PACD** | 0.0220483 | 1.022293 | -0.0260982 | 0.0701949 |
| **Age** | 0.0190903 | 1.019274 | -0.0016873 | 0.0398679 |
| **Elixhauser comorbidity index** | 0.1930107 | 1.212896 | 0.0617039 | 0.3243175 |
| **Frailty score** | 0.1157946 | 1.122765 | 0.0574712 | 0.174118 |
| **Gender** | -0.719912 | 0.4867951 | -1.191087 | -0.2487368 |
| **Intercept** | -5.873976 | 0.0028117 | -7.290627 | -4.457325 |

**Table S11 Final multivariable model for DOS from validation data from 2018-2020**

| Fall and DOS | Beta-Coefficient | OR | 95% Conf. Interval | |
|---|---|---|---|---|
| **DOS** | 0.0380328 | 1.038765 | 0.855245 | 1.261666 |
| **Age** | 0.0485518 | 1.04975 | 0.9838168 | 1.120101 |
| **Elixhauser comorbidity index** | 0.2086366 | 1.231997 | 0.8468074 | 1.7924 |
| **Frailty score** | 0.0644411 | 1.066563 | 0.9048786 | 1.257137 |
| **Gender** | -2.347167 | 0.0956397 | 0.0145744 | 0.6276051 |
| **Intercept** | -7.691481 | 0.0004567 | 1.81E-06 | 0.1153605 |

**Figure S1 ROC curves for accuracy of predicting fall in unadjusted models**

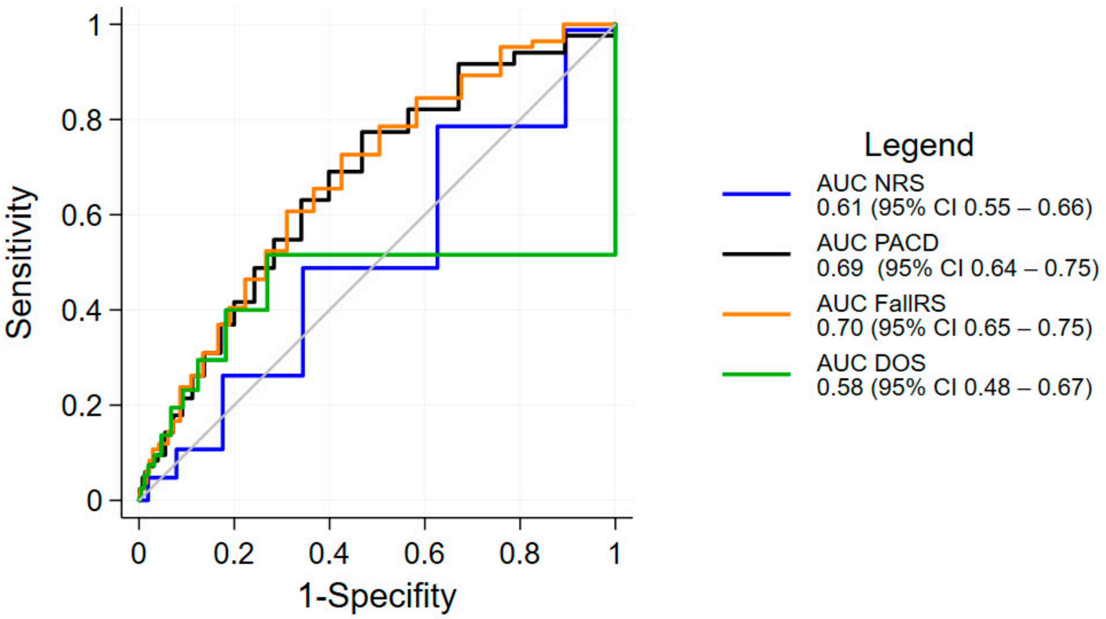


Abbr.: AUC (Area under curve), CI (confidence interval), NRS (nutritional risk screening), DOS (delirium observation screening scale), PACD (post-acute care discharge), FallRS (Fall Risk Score)

**Figure S2 ROC curves for accuracy of predicting fall in adjusted models. Adjusted for age, gender, Elixhauser comorbidity index, Frailty score.**

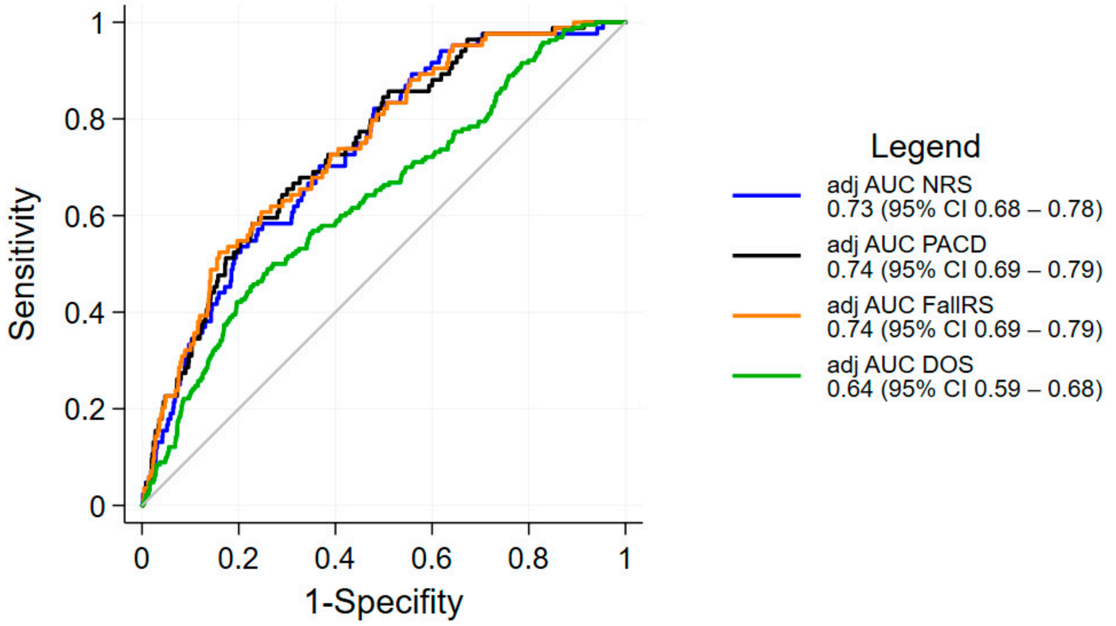


Abbr.: AUC (Area under curve), CI (confidence interval), NRS (nutritional risk screening), DOS (delirium observation screening scale), PACD (post-acute care discharge), FallRS(Fall Risk Score)

**References**

Conca, A., et al. (2018). "Prediction of post-acute care demand in medical and neurological inpatients: diagnostic assessment of the post-acute discharge score - a prospective cohort study." BMC Health Serv Res **18**(1): 111.

Kondrup, J. (2003). "Nutritional risk screening (NRS 2002): a new method based on an analysis of controlled clinical trials." Clinical Nutrition **22**(3): 321-336.

Marieke J Schuurmans 1, L. M. S.-B., Sijmen A Duursma (2003). "The Delirium Observation Screening Scale: a screening instrument for delirium." Research and theory for nursing practice.
